# Adrenaline Auto‐Injectors for Preventing Fatal Anaphylaxis

**DOI:** 10.1111/cea.14565

**Published:** 2024-10-09

**Authors:** Marcus Sim, Vibha Sharma, Karen Li, Mary Hazel Gowland, Tomaz Garcez, Cassandra Shilladay, Richard Pumphrey, Nandinee Patel, Paul J. Turner, Robert J. Boyle

**Affiliations:** ^1^ National Heart and Lung Institute Imperial College London London UK; ^2^ Royal Manchester Children's Hospital Manchester UK; ^3^ Lydia Becker Institute of Inflammation and Immunology University of Manchester Manchester UK; ^4^ Allergy Action St Albans UK; ^5^ Research and Innovation Manchester University NHS Foundation Trust Manchester UK; ^6^ Department of Immunology Manchester University NHS Foundation Trust Manchester UK

**Keywords:** anaphylaxis, drug allergy, food allergy, pharmacology and pharmacogenomics, venom and insect allergy

## Abstract

Anaphylaxis affects up to 5% of people during their lifetime. Although anaphylaxis usually resolves without long‐term physical consequences, it can result in anxiety and quality of life impairment. Rarely and unpredictably, community anaphylaxis can cause rapid physiological decompensation and death. Adrenaline (epinephrine) is the cornerstone of anaphylaxis treatment, and provision of adrenaline autoinjectors (AAI) has become a standard of care for people at risk of anaphylaxis in the community. In this article, we explore the effectiveness of AAIs for preventing fatal outcomes in anaphylaxis, using information drawn from animal and human in vivo studies and epidemiology. We find that data support the effectiveness of intravenous adrenaline infusions for reversing physiological features of anaphylaxis, typically at doses from 0.05 to 0.5 μg/kg/min for 1–2 h, or ~ 10 μg/kg total dose. Intramuscular injection of doses approximating 10 μg/kg in humans can result in similar peak plasma adrenaline levels to intravenous infusions, at 100–500 pg/mL. However, these levels are typically short‐lived following intramuscular adrenaline, and pharmacokinetic and pharmacodynamic outcomes can be unpredictable. Epidemiological data do not support an association between increasing AAI prescriptions and reduced fatal anaphylaxis, although carriage and activation rates remain low. Taken together, these data suggest that current AAIs have little impact on rates of fatal anaphylaxis, perhaps due to a lack of sustained and sufficient plasma adrenaline concentration. Effects of AAI prescription on quality of life may be variable. There is a need to consider alternatives, which can safely deliver a sustained adrenaline infusion via an appropriate route.

## Graphical abstract

During 35 years of increasing Adrenaline Autoinjector prescribing, there has been no obvious reduction in numbers of fatal anaphylaxis cases. Animal and human studies suggest intravenous adrenaline infusion, with appropriate fluid resuscitation, prevents fatal anaphylaxis. Current Adrenaline Autoinjectors may have little impact on rates of fatal anaphylaxis due to a lack of sustained and sufficient plasma adrenaline concentration.

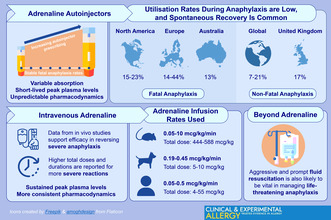

**Summary**

- Adrenaline autoinjectors have been prescribed since 1987, often with the intention to prevent fatal anaphylaxis.
- Animal and human studies suggest intravenous adrenaline infusion, with appropriate fluid resuscitation, prevents fatal anaphylaxis.
- Available data suggest that current adrenaline autoinjectors may have little impact on preventing fatal anaphylaxis.

## 1 Anaphylaxis Management

Anaphylaxis is an acute systemic hypersensitivity reaction to an allergen or trigger, typically associated with airway, respiratory or circulatory compromise. Anaphylaxis is defined by clinical features rather than a diagnostic test, but consensus criteria such as those produced by the World Allergy Organization Anaphylaxis Committee are highly sensitive for detecting anaphylaxis [1, 2, 3, 4, 5, 6, 7, 8, 9]. Anaphylaxis is relatively common, affecting up to 5% of United States and 0.5% of European citizens [10, 11]. Fatal outcome is rare in most settings with a population incidence of 0.03–0.51 per million/year [10], but severity cannot be easily predicted, and anaphylaxis is therefore considered a medical emergency with intramuscular (IM) adrenaline advised as first‐line treatment. To facilitate early adrenaline administration, individuals at risk of anaphylaxis are often prescribed adrenaline autoinjectors (AAI) for self‐administration. The experience of anaphylaxis, and ongoing risk of further episodes, can have a significant impact on mental health and quality of life [12, 13]. In this article, we review the role of adrenaline in anaphylaxis management, and explore the effectiveness of AAI for preventing fatal outcome.

### 1.1 Adrenaline for Treating Anaphylaxis

Adrenaline is an endogenous catecholamine produced by the adrenal medulla in response to exertion or stress. Adrenaline has both alpha‐ and beta‐adrenergic receptor agonist actions [14]. In anaphylaxis, effector cell mediators drive vasodilation, capillary leak and bronchospasm, which are responsible for the main clinical features [15, 16, 17]. Adrenaline's alpha‐adrenergic stimulation of vascular smooth muscle and endothelial cells mediates potent vasoconstriction, mitigating against vasodilation, further capillary leak and hypotension. Beta‐1‐adrenergic stimulation has inotropic and chronotropic effects on the heart, increasing myocardial contractility and heart rate. Beta‐2‐adrenergic stimulation produces bronchodilation, counteracting the bronchospasm and wheeze seen in anaphylaxis. In animal models, adrenaline also inhibits mast cell exocytosis through beta‐adrenergic receptors [18].

IM injection of adrenaline at 0.01 mg/kg (maximum 0.5 mg) into the anterolateral thigh is recommended for managing anaphylaxis, although some guidelines also dose by age with a maximum 0.5 mg > 12 years [19, 20]. This route is associated with higher peak plasma adrenaline concentrations than subcutaneous (SC) adrenaline in healthy, normal‐weight adults [21], but not necessarily in those with increased subcutaneous adiposity [22]. Time to peak plasma adrenaline is also delayed with SC compared to IM [23]. IM adrenaline is generally well‐tolerated, but intravenous (IV) adrenaline can have serious adverse effects [24, 25]. Nevertheless, in refractory anaphylaxis, continuous adrenaline infusion with close cardiovascular monitoring may be needed [26]. Some guidelines also recommend nebulised adrenaline as an adjunct in certain scenarios [2, 20]. Intranasal [27] and sublingual [28] routes have been studied with preliminary reports of comparable pharmacokinetics and pharmacodynamics to IM [29, 30].

Although adrenaline is universally recommended for anaphylaxis management, there is uncertainty about the effectiveness of current adrenaline treatments for preventing fatal anaphylaxis. This is partly due to difficulty studying the rare, unpredictable outcome of fatal anaphylaxis.

### 1.2 Discovery of Adrenaline as a Treatment for Anaphylaxis

**FIGURE 1:**
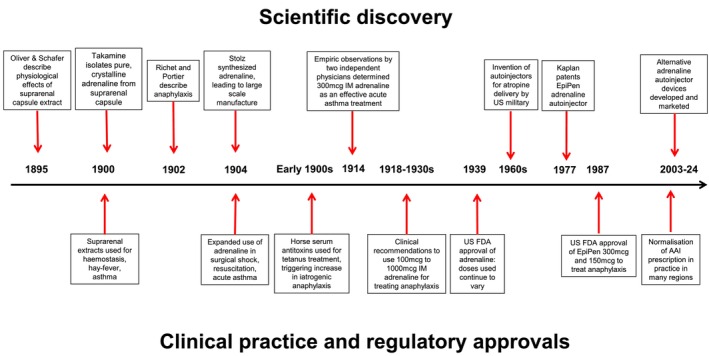
History of adrenaline discovery and adrenaline auto‐injector approval for treating anaphylaxis. Summary of key milestones in adrenaline's discovery, regulatory approval and medical use for treating anaphylaxis.

Oliver and Schafer demonstrated a physiologically active compound from the suprarenal capsules of animals in 1895 (Figure 1), documenting respiratory, cardiovascular and skeletal muscle effects [31]. The arteriolar constriction effect was used by medical professionals for haemostasis [32], hay fever and asthma [33] from 1900. Takamine isolated pure adrenaline with potent pharmacological effects in 1900 [34], and Aldrich identified the chemical formula in 1901 [35]. In 1904, Stolz achieved laboratory synthesis of adrenaline [36], leading to large‐scale manufacturing for use in surgical shock, resuscitation and acute asthma, notably by Parke, Davis & Company [37, 38].

In 1902, Richet and Portier described anaphylaxis in dogs using *Actinia* venom extract [39]. Horse serum antitoxins became widely used to prevent and treat conditions such as tetanus or diphtheria in the early 1900s, with fatal anaphylaxis to antitoxin subsequently described [40]. The link between fatal anaphylaxis and sensitisation was later confirmed by Boughton and Babcock, with asthmatics at increased risk [41, 42]. This improved understanding of anaphylaxis, combined with adrenaline's established role in treating asthma [43, 44] and circulatory arrest [45], contributed to the use of IM adrenaline for anaphylaxis.

The 0.3 mg IM adrenaline dose was independently determined by two separate physicians in 1914 for treating asthma [46], with the same dose recommended for anaphylactic shock by a 1917 American pharmacology guide [47]. In physician guides between 1918 and 1930s, 0.1–1.0 mg IM adrenaline was recommended for anaphylaxis, based on clinical experience [46]. The US Food and Drug Administration (FDA) initially approved adrenaline in 1939 [48, 49] for asthma, hay fever, urticaria, serum sickness and angioneurotic oedema [50, 51]. However, dosing continued to vary until the development of AAIs containing standardised doses for anaphylaxis.

### 1.3 Development of Adrenaline Autoinjectors

Autoinjectors were invented in the 1960s for the US military, enabling timely administration of IM atropine, a nerve agent antidote [52, 53]. They have also been used for rapid administration of benzodiazepines, triptans and naloxone to treat seizures, migraines and opioid overdose, respectively [54]. In 1977, Sheldon Kaplan registered a patent for EpiPen whilst working for Survival Technology Inc [55]. FDA‐approved EpiPen to treat anaphylaxis in 1987 (NDA 019430, EpiPen 0.3 mg and EpiPen Jr. 0.15 mg) [56] and marketing authorisations in Germany (1989) and UK (1996) followed [57]. Despite a long history of adrenaline use, the evidence reviewed for these authorisations did not include any clinical trial data determining optimal dosing or clinical effectiveness for managing anaphylaxis in humans.

EpiPen was the sole AAI until FDA approval of Twinject in 2003 [58]. Currently, there are three name‐brand FDA‐approved AAIs marketed (EpiPen, Adrenaclick and Auvi‐Q) [59]. Within Europe, eight name‐brand AAIs have European Medicines Agency approval [60], the most prescribed being EpiPen, Jext and Anapen. Despite increased competition, EpiPen retained 90% market share worldwide in 2015 [61]. Generic AAIs were first approved by FDA in 2018 [62, 63].

AAIs have a central place in current recommendations for anaphylaxis management, especially in the community, but there are known challenges. These include access and availability (complicated by device cost and expiry), hesitancy of patients, carers and health practitioners to use them, device design limitations and uncertainty about the optimal dose in anaphylaxis [22, 64, 65].

### 1.4 AAI Availability, Prescription Rates and Utilisation

**FIGURE 2:**
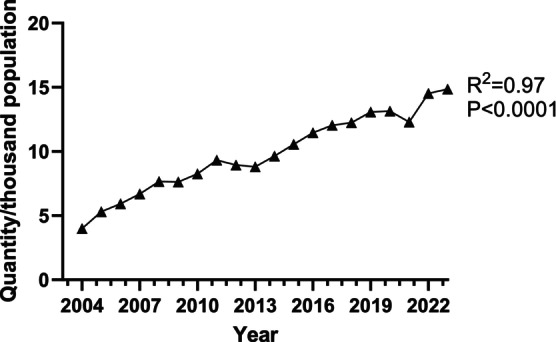
Rise in AAI prescribing in England. Data shown are the number of AAIs prescribed in the public health system (National Health Service, NHS) in England from 2004 to 2023, expressed as quantity per thousand population. Data are for community prescriptions and do not include prescriptions issued in hospital or outside of the public health system. Data were extracted from NHS Business Services Authority for prescriptions, and from the Office of National Statistics for population data. Prescriptions are expressed as number of individual AAI items prescribed each year.

Availability of AAI is not consistent over time or geography. In November 2017, EpiPen faced global supply issues, with shortages reported in Australia, Canada, the US and the UK [64]. Competitor devices such as Jext and Emerade have also faced supply issues [66, 67]. In many regions, AAI are not yet marketed. For example, in 2017 only 32% of 195 countries had access to AAIs, mainly high‐income countries [68]. A 2023 survey confirmed ongoing disparities in global AAI availability [69]. Within high‐income settings with good AAI availability, consistent sales growth is seen [70, 71, 72]. For example, in England, prescriptions dispensed increased at a compound annual growth rate of ~ 9% for the past 20 years (Figure 2). This increase is only partly explained by increased device numbers prescribed to each person at risk of anaphylaxis [73, 74].

AAI prescriptions are not always dispensed, and AAIs are often not carried or used during anaphylaxis [75, 76]. For example, only 226/1538 (14.7%) self‐treated cases in the European Anaphylaxis Registry received adrenaline via AAI [77]. In a further 22.4%, AAI was available but not used, and there was no increase in non‐professional AAI use between 2006–2008 and 2015–2017. Others have also reported low AAI carriage and use in anaphylaxis [78, 79, 80, 81]. The reasons underlying increased AAI prescribing are not clear. AAI marketing, changing healthcare professional behaviours, public perception of risk, increasing food allergy diagnosis, and anaphylaxis awareness may all be relevant.

The efficacy of AAIs in treating anaphylaxis is difficult to study robustly. This evidence gap may contribute to variable AAI availability by impacting on cost‐effectiveness modelling and regulatory approvals [82].

## 2 Evidence That Adrenaline Prevents Fatal Outcome in Anaphylaxis

There is a lack of evidence from randomised controlled trials (RCTs) for the efficacy of adrenaline in anaphylaxis [83, 84]. Key challenges in designing such trials include defining anaphylaxis, and identifying an outcome measure, which is common enough to allow the study to be powered, but important enough to influence practice. Comparisons are sometimes made to the lack of RCT evidence for parachutes; however, death is almost certain without a parachute, whereas anaphylaxis usually resolves spontaneously without treatment [85, 86]. Serious adverse physical outcomes of anaphylaxis, such as fatality [10, 87] or neurodisability [88, 89] are extremely rare. Moreover, the pathophysiology of fatal anaphylaxis is not uniform or well characterised, so that surrogate outcome measures for potentially fatal anaphylaxis are lacking [90]. Therefore, current evidence for adrenaline preventing fatal anaphylaxis relies on epidemiology, animal studies and small observational studies in humans.

### 2.1 Epidemiological Data

Epidemiological studies report that the incidence of anaphylaxis, emergency department (ED) attendance for anaphylaxis, and related hospital admissions have increased in recent decades. The most marked change observed is in childhood food anaphylaxis [91, 92]. However, population rates of fatal anaphylaxis have remained stable over a similar period [10]. The apparent increase in non‐fatal anaphylaxis is likely to be due to changes in public and professional attitudes to allergic reactions, coding, health‐seeking behaviour and diagnostic testing. A marked shift in social responses to allergic reactions is supported by data showing a 4‐ and 7‐fold increase, respectively, in the number of paediatric allergy clinics and new patient appointments in the UK between 2006 and 2019–2020 [93]. If AAI are effective for preventing fatal anaphylaxis, we might expect the increase in concern about anaphylaxis and in AAI prescribing to be accompanied by a fall in population rates of fatal anaphylaxis.

**FIGURE 3:**
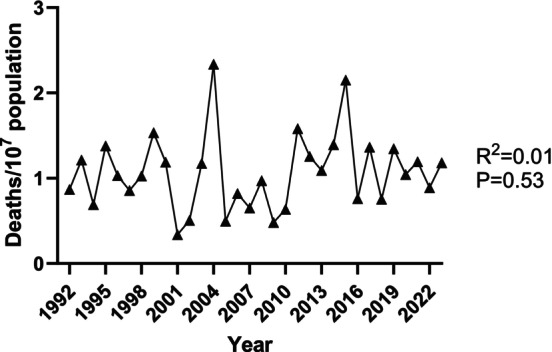
Stable rate of fatal food anaphylaxis in the United Kingdom. Data shown are from the UK fatal anaphylaxis registry [93], expressed as number of fatal food anaphylaxis episodes per 10 million population per annum. Population data were sourced from the UK Office of National Statistics.

In an analysis of England and Wales data for 1992–2012, we found anaphylaxis admissions increased by 7.3% per annum but fatality rates were stable [94]. During the same period, AAI prescriptions increased by 11.3% per annum. In some studies, an increase in fatal drug anaphylaxis has been identified, perhaps due to increasing population exposure to potentially allergenic therapeutics [95, 96, 97, 98]. However, overall rates of fatal anaphylaxis are stable in most studies, despite increased AAI prescribing [99]. Our updated UK data (Figure 3) continue to show relatively stable population rates of fatal food anaphylaxis, despite increased AAI prescribing. These patterns suggest that AAIs are either not being carried/used, or are not effective for preventing fatal anaphylaxis.

### 2.2 Animal Studies: Ovalbumin‐Sensitised Rats

Table 1 summarises adrenaline doses needed to manage experimental anaphylaxis in animal and human studies. Two groups have undertaken RCTs of anaphylaxis management in ovalbumin‐sensitised rats. In a 2013 study by Zheng, every rat randomised to ‘no treatment’ died within 15 min, with profound and progressive decrease in cardiac output (CO) and mean arterial pressure (MAP) [100]. Rats randomised to adrenaline (or methylene blue) all survived. Adrenaline was administered by continuous IV infusion at 10 μg/kg/min, with two 2.5 μg (~ 9 μg/kg) boluses at 3‐ and 5‐min following ovalbumin injection. A femoral artery catheter showed progressive MAP and CO increase with adrenaline, reaching 70% of baseline MAP and 26% of baseline CO at 60 min. Dewachter similarly used continuous adrenaline infusion at 0.05–10 μg/kg/min and two boluses from 5 min (1 and 3 μg/kg) and all treated rats survived, compared with none in the control group [101]. In general, recovery findings were similar between the two trials, although Zheng did not demonstrate recovery of quadricep muscle PtiO_2_ at 60 min whereas Dewachter did, perhaps partly due to the use of larger bolus doses in Zheng's study (~ 9 μg/kg cf. 1–3 μg/kg).

**TABLE 1:** Adrenaline doses and infusion rates for treating anaphylaxis in animals and humans.

Author	Model	Grading	Route	Rate (μg/kg/min)	Total dose (μg)	Total dose by weight (μg/kg)	Total duration of infusion (min)	Clinical outcome
Animal studies								
Bautista et al. (2002)	Ragweed‐sensitised dogs	Hypotensive shock (*n* = 6–11)	IV, IM or SC bolus (*n* = 6, *n* = 9, *n* = 11, respectively, crossover)	—	—	10	—	No difference from placebo in haemodynamic measures at 15 min
Mink et al. (2004)	Ragweed‐sensitised dogs	Hypotensive shock (*n* = 6)	IV, IM or SC bolus or IV infusion (*n* = 6 crossover)	0.19–0.45 (titrated to achieve MAP 70% baseline)	—	10 (bolus) or 5 (infusion)	40	Faster recovery of MAP and CO with infusion No differences between IV, IM, SC bolus and control
Dewachter et al. (2006)	Ovalbumin‐sensitised rats	Hypotensive shock (*n* = 7)	IV bolus (*n* = 7)	—	140.8	444.3	—	All survived with IV adrenaline At bolus doses > 100 μg/kg, 43% developed reversible ventricular tachycardia
Dewachter et al. (2007)	Ovalbumin‐sensitised rats	Hypotensive shock (*n* = 24)	IV bolus and infusion (*n* = 24)	0.05–10.0 (titrated to achieve MAP 60 mmHg)	—	—	54	All survived with IV adrenaline All died in control group
Zheng et al. (2013)	Ovalbumin‐sensitised rats	Hypotensive shock (*n* = 60)	IV bolus and infusion (*n* = 60)	10	161.2	588.3	57	All survived with IV adrenaline All died in control group
Zheng et al. (2015)	Ovalbumin‐sensitised rats	Hypotensive shock (*n* = 27)	IV bolus and infusion (*n* = 27)	10 (titrated to achieve MAP 75 mmHg)	—	—	—	All survived with IV adrenaline All died in control group
Human studies								
Smith et al. (1980)	Adult; sting anaphylaxis	Grade 4 (*n* = 3)^b^	IV bolus (*n* = 3)	—	3830	54.76^e^	—	All survived 2 required multiple IV boluses of adrenaline and recovered only when continuous noradrenaline/adrenaline infusions commenced
Brown et al. (2004)	Adult; sting anaphylaxis	Grade 1 (*n* = 5)^a^	IV infusion (*n* = 19)	0.07–0.21	520*	7.43*^,^ ^e^	92*	All survived Hypotensive reactions received significantly more adrenaline and longer infusions
		Grade 2 (*n* = 3)^a^						
		Grade 3 (*n* = 3)^a^						
		Grade 4 (*n* = 8)^a^			762*	10.89*^,^ ^e^	169*	
Ruiz‐Garcia et al. (2018)	Adult; peanut anaphylaxis	Grade 2–3 (*n* = 14)^b^	IM only (*n* = 14)	N.A.	500	7.14^e^	—	All survived
Harper et al. (2018)	Adult and child; perioperative anaphylaxis	Grade 3 (*n* = 146)^c^	IV only (*n* = 165) IM only (*n* = 37) IM + IV (*n* = 16)	—	200 (IV)^∅^	—	—	10 fatalities (3.8% mortality), 40 cardiac arrests (15%) Median IV total dose was higher in those with more severe anaphylaxis
		Grade 4 (*n* = 110)^c^			500 (IV)^∅^	—		
		Grade 5 (*n* = 10)^c^			4000 (IV)^∅^	—		
Khaleva et al. (2020)	Child; perioperative anaphylaxis	Grade 3 (*n* = 25)^d^	IV only (*n* = 22) IM only (*n* = 3) IM + IV (*n* = 3) IV + INH (*n* = 1)	—	383.7^#^	3.8^#^	—	All survived Respiratory/cardiovascular symptoms significantly associated with increased number of doses required
		Grade 4 (*n* = 4)^d^		—	2735.75^#^	54.2^#^	—	
Fujizuka et al. (2022)	Adult; all‐cause anaphylaxis	Grade 1–3 (*n* = 134)^b^ Grade 4 (*n* = 8)^b^	IV only (*n* = 78)	0.10 (titrated to SBP 90–140 mHg)	250*	3.6*^,^ ^e^	5	All survived Significantly lower total dose and shorter time to symptom resolution with IV adrenaline
			IM only (*n* = 64)	N.A.	300*	4.3*^,^ ^e^	—	

*Note:* Adrenaline doses, infusion rates and durations for treating anaphylaxis in rodent, canine and human studies.

Abbreviations: CO, cardiac output; IM, intramuscular; IV, intravenous; MAP, mean arterial pressure; SC, subcutaneous.

^a^ Müller grading.

^b^ WAO 2024 grading.

^c^ NAP6 grading.

^d^ Ring and Messmer (modified).

^e^ Mean weight assumed 70 kg.

*p < 0.05, ^#^ *p* < 0.01, ^∅^ *p*‐values not available.

These groups conducted other studies, summarised in Table 1 [102, 103]. Total adrenaline infusion dose by weight in these rodent studies was much higher than that used in human studies [104], perhaps due to the severity of induced anaphylaxis with all untreated rodents suffering fatal outcome. Dewachter's 2006 study utilised increasing bolus doses, noting adverse cardiac effects including ventricular tachycardia and hypertension in 43% of rats administered bolus doses > 100 μg/kg. Moreover, the rats became hypotensive again by 30–55 min. These adverse effects and rebound hypotension were not seen by either group with continuous adrenaline infusion using cumulative doses up to 588 μg/kg, resulting in sustained blood pressure recovery. Hence, in rats, continuous infusion appeared to be safest for large cumulative doses of adrenaline. Indeed, continuous infusion prevented fatal outcome in these three studies, in contrast to 100% mortality in untreated rats in the control group, suggesting that all fatal anaphylaxis may be potentially preventable.

Where haemoconcentration was studied, this was present in all treatment groups and not mitigated compared to ‘no‐treatment’ groups, highlighting fluid redistribution as a significant phenomenon even in treated anaphylaxis in this rodent model. Small boluses of up to 5 mL/kg 0.9% saline were administered during these experiments, less than volumes recommended for treating human anaphylaxis, where fluid resuscitation has been associated with symptom resolution and restored stroke volume [105].

### 2.3 Ragweed‐Sensitised Dogs

Induced anaphylaxis in canine studies is less severe, with no fatalities and full haemodynamic recovery in untreated dogs by 180 min. Hence, per kg doses of infused adrenaline were lower than in the rodent studies. Bautista conducted a placebo‐controlled trial of bolus IV, IM or SC 10 μg/kg adrenaline without continuous infusion, in canine ragweed anaphylaxis [106]. IV adrenaline produced a short‐lived, immediate increase in MAP, stroke volume (SV) and pulmonary wedge pressures compared to placebo, but there were no haemodynamic differences 15 min after shock induction. IM and SC doses did not affect cardiovascular parameters at any time‐point compared to placebo, although plasma adrenaline concentrations were raised in the IM group. In contrast to these largely null findings, continuous adrenaline infusion in a similar canine model was reported by Mink to show improved haemodynamic recovery compared with IV, IM or SC bolus adrenaline in a randomised, controlled, crossover study [107]. A transient response to IV bolus 10 μg/kg adrenaline at 5 min was observed (increased MAP only), with no change in cardiovascular parameters with IM or SC boluses compared with placebo. In contrast, continuous IV adrenaline infusion led to recovery of MAP and CO to baseline within 10 and 5 min, respectively, with a sustained response in MAP, CO and SV.

These canine findings suggest that continuous IV adrenaline infusion yields faster and more sustained recovery of cardiovascular parameters following induced anaphylactic shock in dogs, compared with 10 μg/kg IM, SC or IV bolus or no treatment.

### 2.4 Human Studies

#### 2.4.1 Studies of Intravenous Adrenaline in Anaphylaxis Management

Severe allergic reactions occurring in perioperative and allergy research settings with continuous physiological monitoring have shed some light on human anaphylaxis physiology and the impact of IV adrenaline. Fisher reported a case series of 227 people with perioperative anaphylaxis; 205 (90%) had severe hypotension (systolic BP < 40 mmHg), of whom 192 (94%) had clinical vasodilation; 155 (75%) developed cardiovascular collapse [108]. Electrocardiographic changes, mainly supraventricular tachycardias, were common and when measured, right‐sided cardiac filling pressures were usually low. This was likely due to fluid shifts, on the basis of haemoconcentration measurements consistent with an estimated loss of up to 35% (equivalent to 1–1.5 L) in circulating volume seen within 10 min of onset of anaphylaxis. Respiratory physiology of anaphylaxis is less well‐studied [109]. In two small studies of paediatric perioperative anaphylaxis, bronchospasm was common but cardiovascular changes such as hypotension were almost universal [110, 111]. Fisher reported adrenaline to be the most effective first‐line inotrope. He did not report the doses used, but did explicitly recommend treatment by IV infusion, with appropriate monitoring. In a more recent audit of 266 perioperative anaphylaxis reactions in the UK, median total IV adrenaline doses were 200, 500 and 4000 μg for Grades 3, 4 and 5 reactions (adaptated WAO severity scale) (Table 1) [112].

**FIGURE 4:**
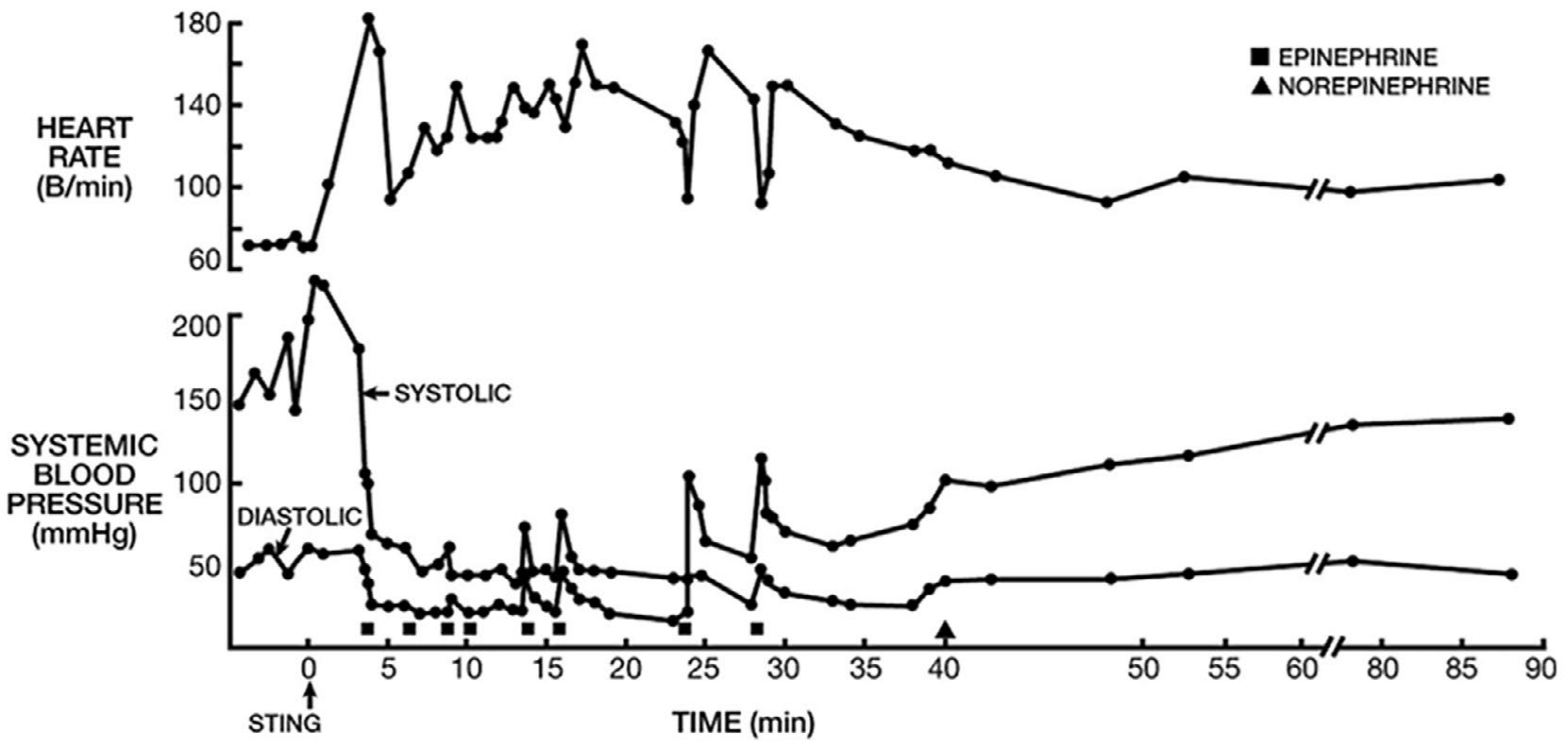
Severe anaphylaxis following an insect sting. Each black square denotes intravenous administration of 500 μg adrenaline as a bolus. The black triangle denotes initiation of a noradrenaline infusion. This case shows a suboptimal and transient response to bolus intravenous adrenaline treatment in an adult undergoing insect sting anaphylaxis. Reproduced with permission from Smith et al. [113].

Brown studied experimentally induced insect sting anaphylaxis and reported management with IV adrenaline infusion [104]. Nineteen adults received infusions, with median total dose and infusion duration of 590 μg and 115 min. Dose and duration were highest for those (*n* = 8) with hypotensive anaphylaxis, at median 726 μg and 169 min and maximum 1310 μg. Five of those with hypotension also received 1000 mL 0.9% saline bolus. Symptoms recurred with adrenaline infusion cessation in 9/19 (47%) people, requiring further infusion. Infusion rates were 0.07–0.21 μg/kg/min, similar to guidance arising from a published case series of moderate severity anaphylaxis, where a starting dose of 0.08 μg/kg/min for moderate anaphylaxis and 0.17 μg/kg/min for hypotensive/hypoxic anaphylaxis was recommended [26]. Smith et al. similarly reported three hypotensive sting anaphylaxis cases managed with IV adrenaline [113]. Much higher cumulative doses (between 4000 and 7000 μg adrenaline) were reported in this study and were associated with only transient rises in blood pressure (Figure 4); however, in contrast to the case series by Brown, adrenaline was given as IV boluses rather than IV infusion. A recent retrospective Japanese study of 142 cases of emergency department (ED) anaphylaxis over a decade [114], 78/142 (55%) cases were managed with low‐dose IV adrenaline infusions and 64 (45%) with 300 μg IM adrenaline. Infusions were commenced at 0.1 μg/kg/min and titrated to maintain SBP at 90–140 mmHg. Those treated with IV adrenaline infusions presented with more severe reactions but required a lower total adrenaline dose (median 250 μg vs. 300 μg), experienced fewer adverse events and were less likely to have a biphasic reaction. Although time to administration was statistically longer in the infusion group (median 7 vs. 4 min), resolution of symptoms was achieved more rapidly (median 5 vs. 9 min). Treatment durations and doses were, however, much lower than those required in prior studies; this is consistent with inclusion of mostly non‐severe reactions, as would be expected for an unselected cohort of anaphylaxis presenting to ED.

The pathophysiology of anaphylaxis described in human studies supports use of adrenaline based on its known, cardiac, bronchodilator and peripheral vasoconstrictor effects, together with adequate fluid resuscitation [14]. These uncontrolled human studies reported much larger adrenaline doses were required for more severe reactions, with 14‐fold greater total IV adrenaline dose by weight for Grade 4 versus Grade 3 anaphylaxis in one study [110] and 8‐ to 20‐fold greater for Grade 5 versus Grade 3/4 in another [112].

#### 2.4.2 Pharmacokinetics and Pharmacodynamics of Intravenous Adrenaline Infusion

**FIGURE 5:**
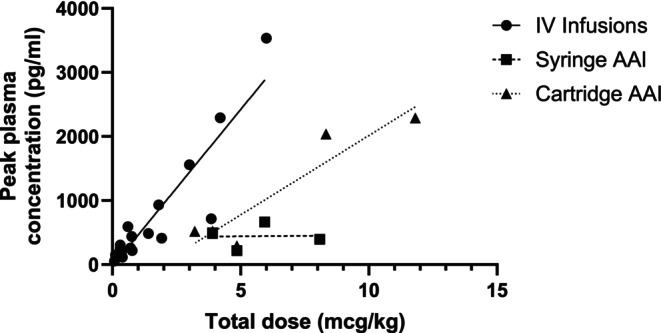
Pharmacokinetics of adrenaline administered via different methods. Data show maximal peak plasma adrenaline concentration in relation to total adrenaline dose administered in human infusion and intramuscular studies [114, 115, 116, 117, 118, 119, 120, 121, 122, 123, 124]. Straight lines are regression lines for each type of adrenaline delivery method. AAI, adrenaline autoinjector; IV, intravenous.

**FIGURE 6:**
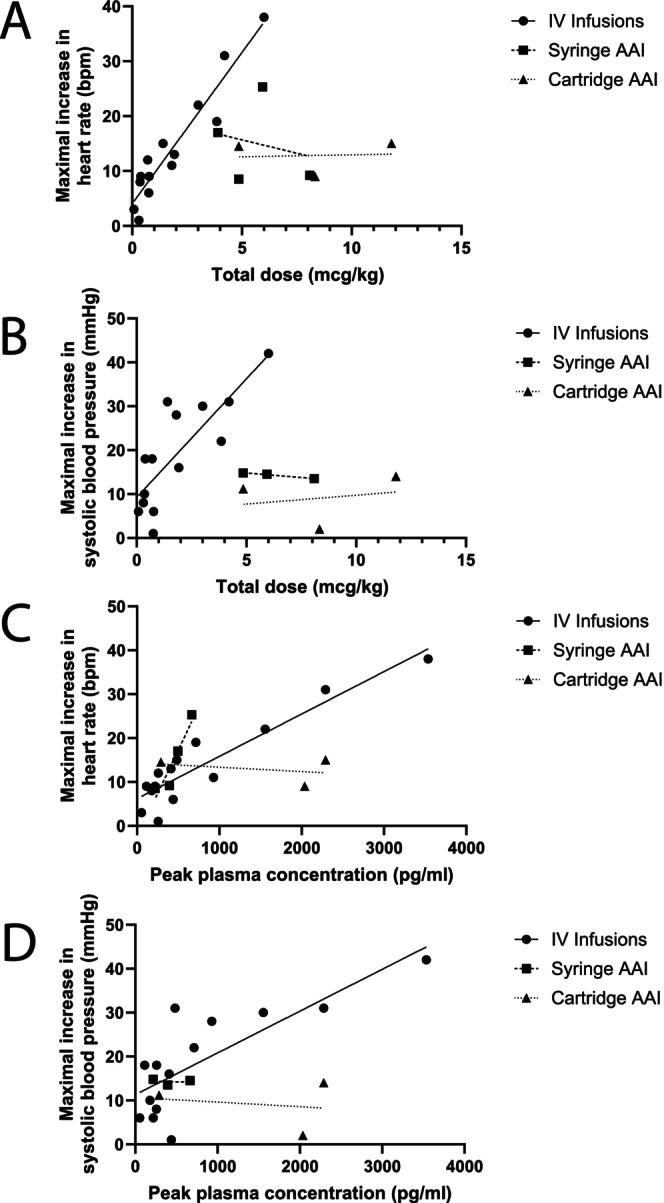
Associations between adrenaline dose or plasma adrenaline concentration and cardiovascular response. Data show maximal increase in cardiovascular parameters with increasing total adrenaline dose (A heart rate, B systolic blood pressure) and with increasing peak plasma concentration (C heart rate, D systolic blood pressure). Data are summarised from studies described in Table 2 [114, 115, 116, 117, 119, 120, 121, 123]. Straight lines are regression lines for each type of adrenaline delivery method. AAI, adrenaline autoinjector; IV, intravenous.

Plasma adrenaline and cardiovascular responses to adrenaline treatment in humans are summarised in Table 2 and Figures 4 and 5. Respiratory responses to adrenaline treatment in humans have not been as well‐characterised. Several studies of healthy volunteers have been undertaken, where more precise evaluation of relationships between dosing, plasma adrenaline and cardiovascular response can be undertaken [115, 116, 117, 118, 125]. Taken together, these studies show strong correlations between adrenaline infusion rates and plasma adrenaline (*R* ^2^ = 0.83, *p* < 0.0001, Figure 5). Moreover, there are strong correlations for both adrenaline infusion rate and plasma adrenaline level with cardiovascular responses such as HR and SBP [115, 116, 117, 118] (Figure 6). These close associations support the importance of precise dosing and continuous cardiovascular monitoring when using IV adrenaline infusions to treat humans. The potential for adverse effects from excessive or inappropriate IV adrenaline means that use of IV adrenaline infusions to treat community anaphylaxis, where fatal outcome is very rare, needs careful consideration. Nonetheless, some guidelines do include low‐dose adrenaline infusions as part of pre‐hospital protocols to treat severe refractory anaphylaxis [20, 126].

**TABLE 2:** Cardiovascular response to adrenaline in human studies.

Author	Route/Device	Rate (μg/min)	Rate by weight (μg/min/kg)	Total dose (μg)	Total dose by weight (μg/kg)	Peak plasma concentration (pg/mL)	HR (bpm)	SV^a^ (%)	SBP (mmHg)	CO^a^ (%)
Studies in healthy volunteers (intravenous)										
Clutter et al. (1980)	Intravenous infusion; *n* = 6 crossover	0.1	0.0013	6	0.08	54 ± 30	+3^∅^	—	+6^∅^	—
		0.5	0.0064	30	0.39	114 ± 28	+9^∅^	—	+18^∅^	—
		1.0	0.0130	60	0.77	219 ± 83	+9^∅^	—	+6^∅^	—
		2.5	0.032	150	1.92	412 ± 89	+13^∅^	—	+16^∅^	—
		5.0	0.054	300	3.85	715 ± 228	+19^∅^	—	+22^∅^	—
Ensinger et al. (1992)	Intravenous infusion; *n* = 16 crossover	—	0.01	—	0.30	256.5 ± 128	+1	—	+8*	—
		—	0.06	—	1.80	930.7 ± 341	+11*	—	+28*	—
		—	0.10	—	3.00	1557.2 ± 271	+22*	—	+30*	—
		—	0.14	—	4.20	2290 ± 311	+31*	—	+31*	—
		—	0.20	—	6.00	3535.8 ± 759	+38*	—	+42*	—
Stratton et al. (1985)	Intravenous infusion; *n* = 10 crossover	—	0.025	—	0.35	178 ± 15	+8*	+26%^#^	+10^#^	+41%^#^
		—	0.05	—	0.70	259 ± 24	+12^#^	+31%^#^	+18^#^	+58%^#^
		—	0.1	—	1.40	484 ± 69	+15^#^	+40%^#^	+31^#^	+74%^#^
Jenn et al. (1991)	Intravenous infusion; *n* = 14	3.87	0.05	58.05	0.75	439.7 ± 71	+6^#^	—	+1	—
Niwa et al. (2006)	Intravenous infusion; *n* = 9 crossover	0.6	0.01	7.2	0.12	151.5 ± 17.8	+1%^a^	+1.9%	–3%^a^	—
		1.5	0.025	17.9	0.30	304.6 ± 34.9	+9.7%*^,^ ^a^	+16.4*	−1.8%^a^	—
		3.0	0.05	35.8	0.60	592.3 ± 62.8	+19.5%*^,^ ^a^	+18.2*	+3.7%^a^	—
Studies in healthy volunteers (intramuscular)										
Simons et al. (2002)	EpiPen Jr.; *n* = 5	—	—	150	8.33	2037 ± 541	+9^∅^	—	+2^∅^	—
	EpiPen; *n* = 5	—	—	300	11.81	2289 ± 405	+15^∅^	—	+14^∅^	—
Duvauchelle et al. (2017)	Anapen; *n* = 30	—	—	300	3.90	496	+17^∅^	—	—	—
Duvauchelle et al. (2022)	Anapen; *n* = 18, BMI 23.3	—	—	500	6.86	750 (95% CI: 598–916)	+22.1^#^	—	+17.7^#^	—
	Anapen; *n* = 18, BMI 29.9	—	—	500	6.23	580 (95% CI: 598–916)	+29.0^#^	—	+14.7^#^	—
	Anapen; *n* = 18, BMI 36.8	—	—	500	5.03	674 (95% CI: 598–916)	+25.2^#^	—	+13.1^#^	—
Patel et al. (2023)	Emerade; *n* = 12 crossover	—	—	500	8.09	394	+9.2 ± 4.4	+30%	+13.5 ± 7.6	+40%
	Emerade; *n* = 12 crossover	—	—	300	4.85	218	+8.5 ± 6.5	+18%	+14.8 ± 13.7	+21%
	EpiPen; *n* = 12 crossover	—	—	300	4.85	290	+14.5 ± 8.3	+3%	+11.2 ± 11.6	+14%
Studies in people undergoing anaphylaxis (intramuscular)										
Ruiz‐Garcia et al. (2018)	21G needle and syringe; *n* = 14	—	—	500	7.14^b^	—	+6.8*	—	+4.8	—

*Note:* Maximal changes in cardiovascular parameters from baseline in human adrenaline studies.

Abbreviations: CO, cardiac output; HR, heart rate; SBP, systolic blood pressure; SV, stroke volume.

^a^ Values only reported as % change from baseline.

^b^ Mean weight assumed to be 70 kg, values displayed as mean ± standard deviation where available.

*p < 0.05 cf. baseline, ^#^ *p* < 0.01 cf. baseline, ^∅^ *p*‐values not available.

#### 2.4.3 Studies of Intramuscular Adrenaline in Anaphylaxis Management

Ruiz‐Garcia evaluated cardiovascular changes during peanut‐induced allergic reactions in adults [105, 127]. Fifty‐seven adults underwent double‐blind, placebo‐controlled food challenge (DBPCFC) to peanut, with 26 undergoing ≥1 further open challenge several months later. A significant decrease in SV was observed during reactions, along with evidence of increased cutaneous blood flow, implying redistribution of intravascular volume towards the skin. A compensatory increase in HR maintained CO during reactions. These changes were independent of reaction severity, but overall reaction severity in the cohort was mild to moderate with no hypotension or shock. In 14 cases of anaphylaxis, treated with 500 μg IM adrenaline via 21gauge needle and syringe, HR increased by median 6.8 beats/min maximum change in first 10 min, with no significant change in SV, MAP or CO [128]. When utilising a composite measure to assess maximal change in SV, HR, MAP and CO in the first 10 min after adrenaline administration, there was an apparent increase in SV, HR and CO [119]. However, in two cases where a second IM adrenaline dose was used, no further change in SV was noted, supporting the need for controlled intervention studies in humans to further explore cardiovascular effects of IM adrenaline in anaphylaxis management. Eight study participants developed similar reactions at re‐challenge but without objective respiratory symptoms, hence not treated with adrenaline. This enabled within‐subject comparison, demonstrating no significant change in SV or CO with IM adrenaline compared to when it was not administered. Only HR showed a significant mean increase of 17 beats/min (95% CI 4.1–29; *p* = 0.016) with IM adrenaline. Thirteen participants had significant respiratory compromise with increased forced expiratory volume in 1 second (FEV1) and peak expiratory flow rate (PEFR) following IM adrenaline administration, but nebulised salbutamol was given at the same time, complicating interpretation.

Taken together, these findings suggest only modest cardiovascular effects and uncertain respiratory effects of a single 500 μg dose of IM adrenaline during peanut anaphylaxis, superimposed on the broader effects of the underlying reaction and associated stress response.

#### 2.4.4 Pharmacokinetics and Pharmacodynamics of Intramuscular Adrenaline

Pharmacokinetic (PK) data are available for most currently available AAIs in Europe and have been recently reviewed [22]. Differences in skin‐to‐muscle depth and AAI design appear to be important factors influencing pharmacokinetic profiles. However, the force and speed [120] of adrenaline injection may play a greater role than needle length. An early PK study in 17 healthy children reported faster peak plasma adrenaline concentration with IM compared with SC adrenaline, usually within 5 min. Subsequently, the same group found no difference in time to peak plasma adrenaline concentration between 150 and 300 μg EpiPen [121]. Figure 3 summarises several IM studies [121, 122, 123, 124, 129, 130], showing a weak and inconsistent pharmacokinetic relationship compared with IV adrenaline infusions, although there may be individual variations in responses to adrenaline [131].

Limitations of PK studies for adrenaline are our poor understanding of the optimal plasma adrenaline range for treating anaphylaxis and individual variation in both the pharmacokinetics of absorption and the physiological response to adrenaline. Many PK studies also assume a uniphasic rather than biphasic absorption profile for IM adrenaline [22]. Pharmacodynamic (PD) studies help to address these issues. As can be seen from Figure 6 and Table 2, there is a weak and inconsistent relationship between IM adrenaline treatment dose and cardiovascular parameters from published studies [121, 122, 123, 129]. Duvauchelle explored some potential explanations for variable PK and PD effects of Anapen in a study of 54 healthy adults [123]. They reported a biphasic increase in plasma adrenaline, consistent with previous findings. Higher body mass index (BMI) was associated with broadening and flattening of the second peak, with corresponding increase in area under the curve, but didn't significantly impact on PD responses. Plasma adrenaline levels were higher than in a prior study using a lower Anapen dose [122].

Patel evaluated PK and PD of AAI devices in the Pharmacokinetics of Intramuscular Adrenaline in Food‐Allergic Teenagers (PIMAT) study [129]. In a randomised, single‐blind crossover trial, 12 well adolescents were monitored whilst self‐administering either Emerade 500 μg, Emerade 300 μg or EpiPen 300 μg, over two separate visits. Ultrasound‐confirmed IM AAI administration caused significantly increased HR, and Emerade AAIs resulted in sustained SV and CO increase. Surprisingly, a negative inotropic effect was seen for EpiPen, with a small but significant decrease in SV and CO, and only a transient HR increase. All three devices showed biphasic absorption profiles, with Emerade 500 producing a higher and more sustained peak plasma adrenaline level compared to Emerade 300. EpiPen 300 μg achieved a faster peak level than the other autoinjectors. The authors hypothesised that the negative inotropic effects of EpiPen may relate to the sharp peak in plasma adrenaline, potentially driving inhibitory G protein‐coupled β2‐adrenoreceptors. Indeed, in 2017, FDA‐approved labelling changes for EpiPen, following reports of rare cases of stress cardiomyopathy following treatment [132]. Takotsubo cardiomyopathy may be caused by endogenous and exogenous catecholamines, with β1‐ and β2‐adrenoceptors potentially implicated in abnormal calcium signalling and subsequent cardiomyocyte injury [133]. Regardless of the mechanism, the PIMAT findings challenge the assumption that rapidity of achieving peak plasma adrenaline levels is desirable for optimum physiological recovery.

#### 2.4.5 Frequency of Adrenaline Use in Fatal Anaphylaxis Cases

Studies of adrenaline treatment for preventing fatal anaphylaxis are limited to retrospective series of fatal or near‐fatal anaphylaxis. These offer some insight into factors associated with fatal outcome and the potential role of adrenaline. There is evidence from small series that delays in adrenaline administration may be associated with fatal outcome in anaphylaxis. Yunginger identified seven cases of fatal food anaphylaxis and adrenaline was not administered immediately in any case [134]. Similarly, a series of four cases of fatal food anaphylaxis in Israel found none had used adrenaline [135]. Several North American case series report 15%–23% of fatalities receiving adrenaline [136, 137, 138, 139]. The UK fatal anaphylaxis registry documents adrenaline given via AAI prior to first arrest in 14%–20% of fatal food anaphylaxis cases, including one where 3 AAIs were administered correctly [90, 91, 92, 93, 94, 95, 96, 97, 98, 99, 100, 101, 102, 103, 104, 105, 106, 107, 108, 109, 110, 111, 112, 113, 114, 115, 116, 117, 118, 119, 120, 121, 122, 123, 124, 125, 126, 127, 128, 129, 130, 131, 132, 133, 134, 135, 136, 137, 138, 139, 140, 141]. In an Australian series of 55 fatal venom or food anaphylaxis cases, 13% received prompt adrenaline at the scene [142]. A French registry cited adrenaline use in 8/18 (44%) fatal anaphylaxis cases, but usually delayed [143, 144]. Community use of adrenaline in non‐fatal anaphylaxis is also infrequent [85], estimated at 7% of adult and 21% of paediatric anaphylaxis in one meta‐analysis [145]. There may be little difference in rate of timely IM adrenaline between fatal and non‐fatal anaphylaxis, so it is unclear how much contribution timely IM adrenaline makes to determining anaphylaxis outcome.

The only direct comparative study, to our knowledge, assessed seven near‐fatal and six fatal food anaphylaxis cases in US children [146]. Adrenaline (route and dose unstated) was administered within 30 min in 6/7 non‐fatalities and within 60 min in 2/6 fatal cases (*p* = 0.103, Fisher's exact test). All survivors received adrenaline prior to or within 5 min of severe symptoms developing, whereas this did not occur in those who died. The two groups had similar rates of other potential risk factors for fatal anaphylaxis, such as reported history of asthma [10].

In summary, current epidemiological data from Canadian, US, UK, Australian and European registries, as well as smaller retrospective case analyses, report minimal use of timely adrenaline in most cases of fatal and non‐fatal anaphylaxis. Most studies suffer from lack of a control group, and a single, small, case–control study provides inconclusive evidence that delayed adrenaline could be a risk factor for fatal outcome in anaphylaxis. Other outcomes associated with AAI for treating community anaphylaxis are not well documented. For example, the little available information on quality of life impact suggests a neutral or negative effect of AAI prescription on health‐related quality of life [147, 148]. This may be because AAI prescription can be perceived as an indicator of greater risk of severe reaction. There may also be other indirect consequences when AAI has been used to treat a reaction, for example Emergency Medical Services may respond differently.

## 3 Conclusions

Randomised controlled trials in animal models show that IV adrenaline infusions effectively prevent fatal anaphylaxis. Indeed, rat studies suggest that every fatal anaphylaxis episode may be preventable, with prompt and adequate treatment. Human studies in healthy volunteers show a predictable, cardiovascular dose–response to IV adrenaline infusions. However, AAIs are associated with variable adrenaline absorption, short‐lived peak plasma levels and unpredictable cardiovascular response. The few published clinical studies in people undergoing anaphylaxis show only limited cardiovascular effects from IM adrenaline and respiratory effects have not been well characterised. The clinical studies suggest that prolonged, continuous IV infusions are required for treating severe anaphylaxis. Fatal anaphylaxis is rare, unpredictable and fast, which makes prevention difficult in practice. Although conditions such as mastocytosis, cardiovascular disease and severe asthma are thought to be risk factors for fatal anaphylaxis, reliable risk stratification is not yet possible [10, 149].

Consistent with the findings from in vivo studies in humans, dogs and rats, the steady increase in AAI availability over the past 35 years has not been accompanied by an equivalent reduction in population rates of fatal anaphylaxis. Thus, AAIs may not be reliably effective for preventing fatal anaphylaxis. Clinical observations document reduced cutaneous and respiratory symptoms with IM adrenaline treatment of anaphylaxis, but these effects have not yet been quantified in a RCT. AAI prescription has effects on quality of life which may be positive, adverse or neutral [147, 148] For effective management of the most severe allergic reactions, adrenaline given by continuous IV infusion, with appropriate fluid resuscitation, is likely to be required—how this is safely achieved in the pre‐hospital setting remains to be determined.

## Author Contributions

M.S.: contributed to project conceptualisation and led project. Undertook analyses, wrote first draft and collated comments from all authors to complete final draft. V.S.: contributed fatal anaphylaxis data, reviewed and commented on manuscript drafts. K.L: contributed prescribing data, reviewed and commented on manuscript drafts. M.H.G.: contributed fatal anaphylaxis data, reviewed and commented on manuscript drafts. T.G.: contributed fatal anaphylaxis data, reviewed and commented on manuscript drafts. C.S.: contributed fatal anaphylaxis data, reviewed and commented on manuscript drafts. R.P.: contributed fatal anaphylaxis data, reviewed and commented on manuscript drafts. N.P.: reviewed and commented on manuscript drafts. P.J.T.: reviewed and commented on manuscript drafts. R.J.B.: conceptualised and supervised project. Reviewed and edited manuscript drafts.

## Conflicts of Interest

M.H.G. has carried adrenaline for emergency use in anaphylaxis since 1978. P.J.T. reports grants from UK Medical Research Council, National Institute of Health and Social Care/Imperial Biomedical Research Centre, UK Food Standards Agency and the Jon Moulton Charity Trust; personal fees from UK Food Standards Agency outside the submitted work. R.J.B. reports payments for editorial work from Wiley, Cochrane and the British Society for Allergy and Clinical Immunology, for expert witness work in legal cases related to food anaphylaxis and infant formula health claims, and for contributing to a report for the World Health Organization; and grant support from the American Academy of Dermatology and the National Institute of Health and Social Care.

## Data Availability

The data that support the findings of this study are available on request from the corresponding author. The data are not publicly available due to privacy or ethical restrictions.
